# The Impact of Urbanization on the Relationship between Carbon Storage Supply and Demand in Mega-Urban Agglomerations and Response Measures: A Case of Yangtze River Delta Region, China

**DOI:** 10.3390/ijerph192113768

**Published:** 2022-10-23

**Authors:** Yinan Yang, Jing Li, Li Wang, Zihao Wang, Yun Ling, Jialong Xu, Chenxin Yao, Yiyan Sun, Yuan Wang, Lixia Zhao

**Affiliations:** 1 School of Geography and Tourism, Anhui Normal University, Wuhu 241002, China; 2 China Railway Shi Dai Architectural Design Institute Co., Ltd., Wuhu 241000, China; 3 Neweco Design Co., Ltd., Shanghai 200433, China; 4 East China Sea Ecological Center, Ministry of Natural Resources (MNR), Shanghai 201206, China; 5 Key Laboratory of Marine Ecological Monitoring and Restoration Technology, Ministry of Natural Resources (MNR), Shanghai 201206, China; 6 Key Laboratory of Ocean Space Resource Management Technology, Ministry of Natural Resources (MNR), Hangzhou 310012, China

**Keywords:** YRD, urbanization, CSD, response measures

## Abstract

Rapid urbanization in mega-urban agglomerations disturbs the balance of carbon storage supply and demand (CSD) and constrains the achievement of sustainable development goals. Here, we developed a socio-ecological system (SES) framework coupled with ecosystem services (ES) cascade and DPSIR model to systematically analyze the impacts and responses of urbanization affecting CSD. We quantified urbanization and CSD using multi-source remote sensing data, such as land use and night lighting, together with related socio-economic data, such as total energy consumption, population and GDP. We found that from 2000 to 2020, the urbanization of Yangtze River Delta region (YRD) led to a decrease of 2.75% in carbon storage supply and an increase of 226.45% in carbon storage demand. However, carbon storage supply was still larger than carbon storage demand, and the spatial mismatch of CSD is the most important problem at present. Therefore, it is necessary to explore the response measures from the comprehensive perspective of SES. We identified key ecological conservation areas using a Marxan model to protect the carbon storage capacity in ecological subsystems, and promoted a carbon compensation scheme based on both the grandfather principle and the carbon efficiency principle, reconciling the contradiction between ecological conservation and socio-economic development in the social subsystem. Finally, this study quantified the threshold of urbanization based on the carbon neutrality target at which CSD reaches an equilibrium state. This study proposed a SES framework, and a set of methodologies to quantify the relationship between urbanization and CSD, which will help mega-urban agglomerations to promote harmonious development of urbanization and ecological conservation and to achieve the carbon peak and carbon neutrality targets proposed by the Chinese government.

## 1. Introduction

A mega-urban agglomeration is a highly integrated urban agglomeration with compact spatial organization and strong economic ties. Fang, et al. (2017) suggested identifying an urban agglomeration based on following characteristics: (1) it had at least three large cities with populations exceeding twenty million, and one of them had more than five million urbanities; (2) its per capita GDP was over $10,000; (3) the proportion of non-agricultural population exceeded 50%, and non-agricultural industries comprised over 70% of the GDP; (4) the GDP centrality of the core city exceeded 45%; (5) its ratio of dependency on exports was over 30%, and its economic density was over 15 million RMB ($2.5 million)/km^2^; and (6) it presented obvious economic belts with half-hour, one-hour and two-hour transportation radii [1]. An urban agglomeration could be defined as a mega-urban agglomeration when its area exceeds 50,000 km^2^, its total population exceeds 50 million and its economic output accounts for more than 10% of the country’s output. China proposes to build five mega-urban agglomerations, including the Yangtze Delta urban agglomeration, the Pearl River Delta urban agglomeration, the Jing-Jin-Ji urban agglomeration, the Yangtze River Middle-Reach urban agglomeration, and the Cheng-Yu urban agglomeration [2]. Within the agglomerations, cities could break through the constraints of the administrative system and develop close common economic and interest-based communities, which have become the strategic areas for economic development and the main areas of new urbanization. However, with urbanization, mega-urban agglomerations are facing increasingly intense conflicts between socio-economic development and ecological protection [2]. One of the most severe problems has been the imbalance between carbon storage supply and demand (CSD) [3]. Carbon storage supply refers to the ability of an ecosystem to absorb atmospheric CO_2_ and store it; the definition of carbon storage demand remains controversial; here, we defined the carbon storage demand by considering total carbon emissions of carbon sources, from the perspective of carbon neutrality. A carbon source refers to an ecosystem that emits CO_2_ to the atmosphere and thereby increases the concentration of CO_2_ in the atmosphere [4]. On the one hand, rapid urbanization has led to a decreased carbon storage supply capacity through the expansion of imperviousness and the conversion of natural landscapes into built-up areas [4,5]. On the other hand, the aggregation of people and economic activities in mega-urban agglomerations has led to a significant incremental increase in carbon emissions and thus to increased carbon storage demand [6]. It has been reported that urban areas, which cover just 2% of the world’s land, have contributed 75% of global carbon emissions [6]. The imbalance between supply and demand for carbon storage due to rapid urbanization is a serious constraint on the effort to achieve carbon neutrality targets [7]. Carbon neutrality is a balance between carbon emissions from fossil fuel use and land use change and carbon sequestration through ecosystems and other technological means [8]. More than 100 countries worldwide have already committed to carbon neutrality targets, and in 2020, the Chinese government announced to achieve its carbon peak target by 2030 and carbon neutrality target by 2060. Meeting the carbon neutrality target could serve to control the rise of global temperatures and the consequent socio-economic and environmental problems caused by increasing concentrations of greenhouse gases. The two determinants of achieving carbon neutrality are increasing carbon storage and reducing carbon emissions [8], corresponding to improving carbon storage supply and limiting carbon storage demand, respectively. Rapid urbanization has contributed to global warming since the Industrial Revolution, while making people much vulnerable to climate change. The increased urbanization and stronger human interference with nature within mega-urban agglomeration has led to an imbalance in CSD, making it more difficult to achieve carbon neutrality targets in mega-urban agglomeration, and there is consequently an urgent need to understand the impact of urbanization on CSD and develop effective response measures to help achieve carbon neutrality targets.

**Figure 1:**
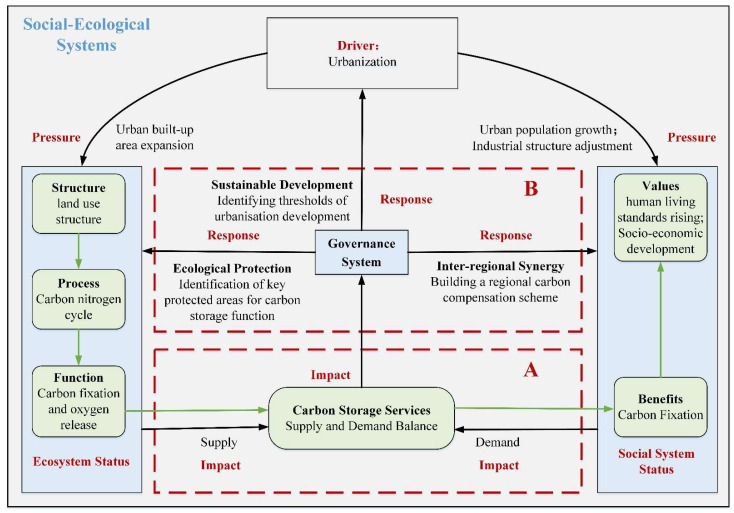
Theoretical framework of social-ecological systems.

The relationship between urbanization and CSD has become a hot topic in the research on interaction between anthropogenic activities and ecosystems. At present, most research has focused on the impact of urbanization on CSD [9]. It has been reported that urbanization has affected carbon emissions in multifaceted and complex ways [10]; such effects could be positive [11], negative [12], offering no significant effect [13], or described as a “hump-shaped” relationship [14]. Urbanization refers to a series of changes, such as population shift, land use change, lifestyle change and industrial restructuring, all of which might affect carbon emissions in different ways [14,15,16,17]. Furthermore, some research has focused on the impact threshold of urbanization on carbon emissions to find the optimal level of urbanization for sustainable development [18]. Additionally, the impact of urbanization on carbon emissions could be varied at global [19], regional [20] and local scales [21]. Another important research topic is about the effects of urbanization on carbon storage supply. Land-use changes caused by urbanization have altered the ecosystem-structure of urban areas and thus affected carbon cycling processes, making research on the impact of urban land-use change on carbon storage a hot issue [22]. It has been reported that reducing above-ground vegetation biomass due to the expansion of impermeable surfaces has led to a loss of carbon storage, which in turn caused a permanent reduction in soil carbon storage potential because of decreased below-ground biomass and carbon density of dead organic matter [23]. It has also been suggested that urban ecosystems provide massive carbon storage with concrete buildings in cities having a strong carbon sequestration capacity and urban soils having a high carbon density [3]. In addition, other important characteristics of urbanization, such as the spatial variation of urbanization and the agglomeration patterns and intensity of spatial expansion also have had significant impacts on carbon storage. However, existing studies have mainly explored the one-sided impact of urbanization on carbon storage supply or carbon storage demand, without integrating the impact of urbanization from both supply and demand perspectives [24]. Along with the intensification of human activities, human society and natural ecosystems have closely interacted to form social-ecological systems (SES), where external forces, or “drivers”, can affect both human society and natural ecosystems. Considering the natural and social attributes of CSD, it is essential to explore the impact of urbanization drivers on CSD from a systemic perspective. Hence, the social-ecological system framework coupling the ecosystem service (ES) cascade and DPSIR model was introduced in this research (Figure 1). The ES cascade has been widely used to link ecological aspects with human well-being, while neglecting societal processes, whereas the DSPIR framework assumes a chain of casual links starting with ‘driving’ forces through ‘pressure’ to ‘states’ and ‘impacts’ on health and functions of natural and social ecosystems, and further leading to ‘response’.

YRD is one of the five mega-urban agglomerations in China, with an area of about 358,000 km^2^. According to the National Bureau of Statistics, in 2020, YRD will have a GDP of 24.5 trillion yuan, accounting for 24% of China’s GDP, and a population of 240 million, accounting for 17% of China’s population. Rapid urbanization has brought great changes in land use structure in this region, leading to a decline in carbon storage supply capacity. Moreover, rapid urbanization and development of social economy has inevitably resulted in increases in energy consumption. It has been estimated that energy consumption of YRD increased twofold during the past 20 years; with the 34.31% of Chinese energy consumption in 2020 making YRD an important source of carbon emissions in China (National Bureau of Statistics), which has also exacerbated the imbalance between carbon supply and carbon demand. In order to eliminate such imbalance, it is essential to quantify the effect of urbanization on CSD, and to promote response measures immediately. Therefore, we quantified the spatial and temporal characteristics of urbanization and CSD in YRD during the years 2000–2020, explored the relationship and intensity of urbanization on CSD, and developed governance response measures for the social subsystem, ecological subsystem and urbanization drivers, respectively. This study intended to: (1) identify the key protected areas for carbon storage functions that need to be protected in YRD, (2) provide a reference to develop inter-regional carbon compensation schemes, and (3) clarify the thresholds for sustainable urbanization. We hope that the study can provide a reference for the coordinated development of urbanization and CSD in mega-urban agglomerations, and promote the achievement of carbon peaking and carbon neutrality in the YRD.

## 2. Theoretical Framework

With the increasing disturbance of anthropogenic activities on ecological processes, human social systems and natural ecosystems have become coupled together, forming SES with the nature of complexity, non-linearity, uncertainty and multi-layered nesting [25,26]. SES reflected the interactions and dynamic feedback between social and ecological subsystems, and was once considered an important analytical framework for promoting harmonious and sustainable development of the ecological environment and socio-economy [27]. However, the description of the process of transferring ecosystem services and goods from the ecosystem subsystem to the social subsystem was lacking in the SES framework, so the ES cascade was created. The ES cascade connects the structure, processes, functions and services of natural ecosystems at one end, and the interests, values and well-being of human social systems at the other end [28,29]. The ES cascade connects the social subsystem and the ecological subsystem well, and describes the transmissive process between nature and human society. However, the ES cascade ignores human participation, and increasingly intense human activities will have an impact on the ES cascade process and upon SES. The DPSIR model is effective in identifying and describing the disturbances and impacts of external factors on systems. Therefore, we embedded the DPSIR model and ES cascade in the SES framework [30] to analyze the impact of urbanization on CSD and response governance (Figure 1). Firstly, blue boxes represent SES elements, while the green boxes represent ES cascade elements, and the arrows represent flow processes. The ecological subsystem is the supply side of ES, and the social subsystem is the demand side of ES. Secondly, when embedding elements of the DPSIR model into SES framework, ‘driving’ forces could exert ‘pressure’ simultaneously on both the ecological subsystem and the social subsystem. ‘State’ alteration has resulted from driving forces’ effects (‘impact’) upon the ES supply and demand through the flow of ES cascade, thus changing CSD throughout the ecosystem. In order to ensure the balance between carbon storage supply and demand, ‘response’ measures need to be developed, including responses for the ecological subsystem, the social subsystem and the urbanization driving force. In this study, we focus on two main issues: the impact of urbanization on CSD (Figure 1, red box A), and responsive governance measures to facilitate the reconciliation of urbanization and CSD (Figure 1, red box B).

In terms of impact research, urbanization has exerted pressures on ecological subsystems through rapid expansion of urban built-up areas during spatial urbanization. This pressure has led to the reduction of natural landscape area, which directly affected the carbon and nitrogen cycling processes in terrestrial ecosystems, resulting in decreased carbon sequestration and limits upon the oxygen release function of the ecological subsystem. We defined such function-decline as a decrease in the ability of the carbon storage supply mechanism. The increment of carbon storage demand is mainly caused by increasing carbon emissions during rapid urbanization. First, urbanization exerted pressures on social subsystems through the transfer of rural population to cities in the process of demographic urbanization, and the shift in industrial structure from primary to secondary and tertiary industries during economic urbanization. Secondly, demographic urbanization led to increased demand for goods consumed by humans in urban areas, which could raise carbon emissions. Besides, the restructuring of industries during economic urbanization, and especially the increased proportion of secondary industries, has led to rapid increases in energy consumption. To meet the demands for values of improved human living standards and socio-economic development, stakeholders in the social subsystem urgently require the ecological subsystem to absorb and bury more CO_2_. Increased demand from the social subsystem and decreased supply from the ecological subsystem had led to the mismatch within CSD. Therefore, in terms of impact studies we wanted to understand the characteristics of the impact of different urbanization indicators on CSD, firstly based on linear regression methods to explore whether different urbanization indicators have an impact on CSD, and secondly based on random forests to explore the intensity of the impact of different urbanization indicators on CSD.

In terms of responsive management measures, the core response of ecological subsystem is ecological conservation. Large amounts of CO_2_ have been released into the atmosphere in the process of urbanization. To ensure that this CO_2_ can be neutralized, it is essential to improve the carbon storage supply ability of ecological subsystems. Here, we used a Marxan model to identify priority conservation areas based on system conservation planning theory to ensure the ability of carbon emissions compensation in YRD. The core response of the social subsystem is to promote inter-regional coordination of ecological conservation and socio-economic development, due to the significant differences in urbanization levels and CSD across the Yangtze River Delta. Therefore, a comprehensive carbon compensation scheme is constructed, based on both the grandfather principle and the carbon efficiency principle. The core response to the drivers of urbanization is urbanization and CSD sustainability. Therefore, based on the CSD equilibrium point, we used a fitted regression model to determine a threshold for urbanization that could help policymakers to reasonably plan the urbanization process in YRD, and achieve coordinated and sustainable development between ecological conservation and socio-economic development.

## 3. Materials and Methods

### 3.1. Study Area

**Figure 2:**
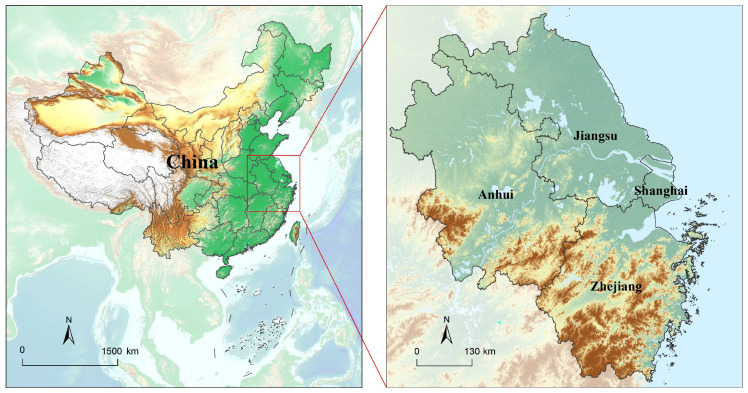
Location of the mega-urban agglomeration-Yangtze River Delta region, China (YRD).

According to the Outline of the Plan for Integrated Regional Development of the Yangtze River Delta released in 2019, YRD covers Shanghai, Jiangsu, Zhejiang and Anhui provinces; its area is approximately 358,000 km^2^ (Figure 2). YRD is a mega-urban agglomeration comprising three mega-cities, Shanghai, Hangzhou and Suzhou, each with populations over ten million, and twenty-nine large cities with populations of more than three million. YRD is the most important economic center, with the fastest urbanization and socio-economic development in China, accounting for about 25% of China’s GDP. The urbanization rate of its resident population exceeds 60%. As one of the largest mega-urban agglomerations in China, the YRD is required to transfer its ecological advantages into economic and social development advantages, and to provide a demonstration of integrated ecological and green development on the premise of strictly protecting the ecological environment. Hence, it is essential to disentangle and quantify the relationships among urbanization, ecological protection, and economic development.

### 3.2. Data Sources

Population density data was derived from WorldPOP (https://www.worldpop.org/geodata/listing?id=64, accessed on 27 November 2021); GDP density data was derived from the inversion of the regression relationship between GDP and land use and night lighting data for each city in YRD. Related calculation process can be found in Methods S1 [31]; the proportion of urban built-up area was counted using the regional statistics function in ArcGIS10.8. Land use data were obtained from the Resource and Environment Science Data Centre of the Chinese Academy of Sciences (https://www.resdc.cn/data.aspx?DATAID=335, accessed on 20 November 2021); urban land use data were obtained based on the Globeland30 land use dataset extracted from man-made surface types. Night lighting data was collected from National Tibetan Plateau Scientific Data Centre [32]. Data on total energy consumption (standard coal) were obtained from the Statistical Yearbooks of Shanghai, Jiangsu, Zhejiang and Anhui. All datasets were calculated on 1 km × 1 km resolution.

### 3.3. Combined Urbanization Level (CUL)

Urbanization refers to a series of changes in terms of the social, economic, cultural and natural landscapes with an increase in the urban population. We chose population, economic and spatial urbanization to create a CUL index, based on the meanings of urbanization [33]. For population urbanization, the urbanization process will lead to a concentration of rural population in urban areas, resulting in an increase in population densities in urban areas. On the one hand, the increase in urban population will increase the total energy consumption and therefore, the carbon emissions; on the other hand, the increase in population density will intensify human interference with nature, thus affecting the carbon storage function. Scholars generally use population density (POP) as an indicator of population urbanization [34,35,36]. For economic urbanization, the process of urbanization promotes a general increase in economic levels, which on the one hand requires a large amount of energy and thus carbon emissions, and on the other hand requires various raw materials provided by nature, thus causing damage to natural ecosystems. Scholars generally use GDP density (GDP) as an indicator of economic urbanization [34,35,36]. The expansion of urban built-up areas is an indicator of spatial urbanization, where urban construction changes land use and thus reduces the capacity of natural ecosystems to store carbon [34,35,36,37,38]. Therefore, artificial land proportion (ALP) is used as the indicator of spatial urbanization. We normalized POP, GDP and ALP and summed them with equal weights to obtain CUL. 

### 3.4. Carbon Storage Supply and Demand 

Carbon storage supply was calculated by land use and carbon density datasets using the carbon storage module in the InVEST model. The carbon density datasets were derived from the existing literature (Table S1). It has been reported that both biomass carbon density and soil carbon density were significantly correlated with mean annual precipitation, and weakly correlated with mean annual temperature [39,40,41]. Therefore, the biomass carbon density and soil carbon density of YRD were corrected by mean annual precipitation variations between China and YRD (Methods S2, Table S2) [39,42].

Carbon emissions were counted using the IPCC’s proposed energy consumption-based carbon emission factor method [43] (Methods S3). Carbon emissions mapping was then developed, based on the relationship between land use and carbon emissions of three industries (Methods S3). Carbon emissions of primary industries were equally allocated to arable land. Carbon emissions from secondary and tertiary industries were inferred by constructing a regression model between nighttime lighting and carbon emissions within urban, rural, industrial, mining and residential land use types. The total carbon emissions of each industry were determined according to the proportion of total energy consumption of primary, secondary and tertiary industries in YRD [31].

Carbon storage supply and demand ratio (CSDR) is defined by Equation (1). It reflects surplus, balance, or deficit of CSD in YRD.

(1) $$CSDR=\frac{S-D}{S+D}$$

where *S* and *D* represent carbon storage supply and carbon storage demand respectively. Positive CSDR indicates surplus of CSD; Negative values indicate deficit of CSD; and 0 indicates a balance of CSD.

### 3.5. Quantify the Impact of Urbanization on CSD

In order to explore the influence of urbanization on CSD, we first constructed a regression model between different urbanization indicators and CSDR based on one-dimensional linear regression; R^2^ indicated whether urbanization indicators have a significant influence on CSDR, and the slope could then be used to compare and analyze the relationship between the influence of different urbanization indicators on CSDR and the difference of the degree of influence. Among them, the max-min method was used to standardize the POP and GDP indicators to ensure the consistency of the quantitative values. Second, random forest regression analysis was used to explore the strength of the effects of different urbanization indicators on CSDR and to understand which urbanization indicators contributed more to the variation of CSDR. Random Forest is a decision-tree-based machine learning algorithm for classification and regression analysis [44]. The random forest regression model could explain the influence degree of independent variables on dependent variables, where sensitivity represents the contribution of independent variables to the accuracy of the random forest regression model. Therefore, with CSDR as the dependent variable and POP, GDP and ALP as the independent variables, sensitivities of CSDR to urbanization characteristics were analyzed through a random forest regression model to reflect the importance of each urbanization indicator affecting CSD change. Related statistics were carried out using the Random Forest package in R Studio software, with IncMSE values characterizing the sensitivity characteristics. For details, see Methods S4.

### 3.6. CSD Response to Urbanization

(1) Identification of priority conservation areas for carbon storage supply using Marxan model.

The Marxan model is a conservation planning decision-aid model developed based on the theory of systematic conservation planning. It calculates the irreplaceability values of planning units through a simulated annealing algorithm and identifies planning units with high irreplaceability as priority conservation areas. Based on this model, priority conservation areas for carbon storage supply that meet the ecological conservation objectives with relatively low ecological conservation costs can be identified. In this study, the CUL represents conservation costs, where higher CUL indicates higher intensity of anthropogenic activities, and greater difficulty in turning the area into a protected area. For the conservation target, two scenarios were set, based on the current year (2020) and the year that carbon peaking target is scheduled to be achieved (2030), and the conservation target was set as the proportion of carbon stock supply to the total carbon stock supply of YRD when CSD reaches the equilibrium point in each scenario. The conservation targets of 2020 and 2030 scenarios are 0.12 and 0.22, respectively. Finally, we obtained an irreplaceability value of each planning unit after running the Marxan model 10,000 times. Irreplaceability value refers to the number of times a planning unit is selected as a priority conservation area, and higher value indicates more important the conservation area is. Combining irreplaceability values with conservation objectives, the priority conservation areas, consisting of planning units with irreplaceability values greater than 2788 and 3421 times in the 2020 and 2030 scenarios respectively, were set to be thresholds to achieve balanced CSD in YRD. For details, see Methods S5.

(2) Building a carbon compensation scheme based on the grandfather principle and the carbon efficiency principle.

**Figure 3:**
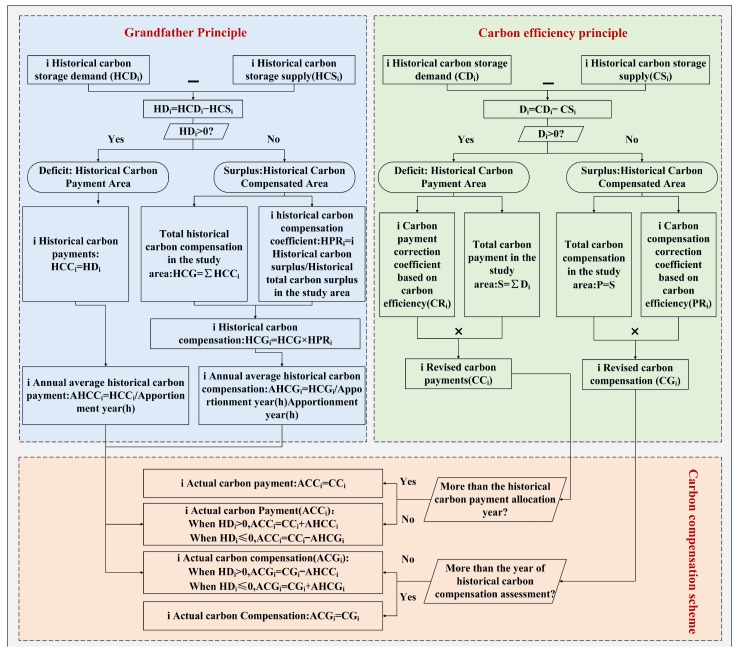
Carbon compensation scheme consisting of the grandfather principle and the carbon efficiency principle.

Accurate accounting of carbon compensation is vital for establishing carbon compensation mechanisms. The existing carbon compensation accounting is mainly based on the carbon deficit or carbon efficiency principle [45,46], but ignores the historical carbon emission differences of each region. Therefore, this study combined the grandfather principle and the carbon efficiency principle to construct an inter-regional carbon compensation scheme (Figure 3).

The YRD were divided into historical carbon payment area and historical carbon compensation area, according to the deficit and surplus of CSD of each region over the historical period. In the historical carbon payment area, the total amount of historical carbon payment was accounted and apportioned to a certain number of years to obtain the average annual historical carbon payment. In the historical carbon compensation area, the historical carbon compensation was determined based on the proportion of the historical carbon surplus to the total historical carbon surplus of the YRD, and was spread over a certain number of years to obtain the average annual historical carbon compensation for each region. In this study, we defined 2000–2020 as the accounting period for historical carbon liability, and the amortization year was set to be 10 years.

The amount of carbon payment was adjusted through carbon efficiency to encourage low-carbon urbanization. Based on the characteristics of urbanization, the carbon efficiency index was constructed using carbon emissions per unit area, carbon emissions per unit GDP and carbon emissions per unit of population. With the higher carbon efficiency, the lower the carbon payments or the higher the carbon compensation. The carbon efficiency correction factors CR and PR were calculated in Methods S6.

(3) Identification of urbanization development thresholds through regression model.

It is reported that urbanization in YRD has a significant negative correlation with CSDR, indicating that increasing urbanization led to a gradual imbalance of CSD. To keep the balance of CSD in YRD and to facilitate the achievement of carbon neutrality targets, it is necessary to identify thresholds of CSD response to urbanization [34]. Therefore, based on the principle of best fit, a stepwise regression was used to select the best fit regression equation for the CSDR (y) and urbanization target (x) [47], and the carbon neutrality target (CSDR = 0) was used to identify the urbanization response thresholds.

## 4. Results

### 4.1. Urbanization and CSD Characteristics

#### 4.1.1. CUL of YRD

YRD had achieved rapid urbanization during 2000–2020, with the average CUL rising from 0.03 to 0.06. Economic urbanization contributed most to increased CUL, with GDP rising by 980.13%, followed by spatial urbanization and demographic urbanization, with ALP and POP rising by 85.71% and 17.78%, respectively.

**Figure 4:**
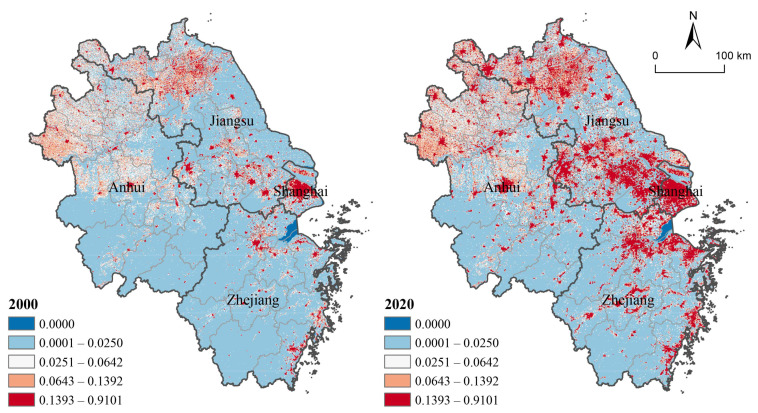
CUL of YRD during 2000–2020.

The urbanization level of YRD showed obvious spatial differentiation. Specifically, high CUL areas manifested in three patterns: point, belt and planar distribution (Figure 4). The point distribution refers to the gradient expansion outward with a single city center as the core, such as Hefei. The belt distribution refers to highly urbanized areas along rivers or coasts which are connected into bands, mainly including the zone along the Yangtze River, Hangzhou Bay and Zhejiang coastal area and the zone between Shanghai, Hangzhou, and Jinhua. The planar distribution refers to the concentration of highly urbanized areas appearing in patches. There were three planar distribution areas, including the Shanghai-Suzhou-Wuxi-Changzhou area, northern Jiangsu and northern Anhui. 

High spatial variation of urbanization could be found among all provinces of YRD (Figure 4). Shanghai had the highest CUL and a broader distribution of highly urbanized areas showing a typical planar distribution. The CUL of Jiangsu was second only to Shanghai, and the spatial distribution of its CUL was relatively uniform, showing a combination of planar and point patterns. The CUL of Zhejiang province was lower, but increased rapidly with a 204.31% increment from 2000 to 2020. Its urbanization was characterized by centralization, with highly urbanized areas densely distributed in the plains along Hangzhou Bay, Fuchun River and the southeastern coastal areas, which was a typical belt distribution. Anhui had the lowest CUL and the slowest urbanization, and its CUL increased 61.23% during the past 20 years.

#### 4.1.2. CSD of YRD

From 2000 to 2020, carbon storage supply in YRD was able to meet its total demand, and there was an overall surplus of CSDR. However, the surplus of CSDR in YRD tended to decrease, with the CSDR decreasing from 0.91 in 2000 to 0.74 in 2020. Compared with 2000, carbon storage demand in YRD had increased by 226.45% in 2020, while meanwhile, its carbon storage supply had decreased by 2.75%. Hence, rapid increased demand of carbon storage caused by massive carbon emissions from the economic and population boom might be the major casualty for the deficit of YRD CSD.

**Figure 5:**
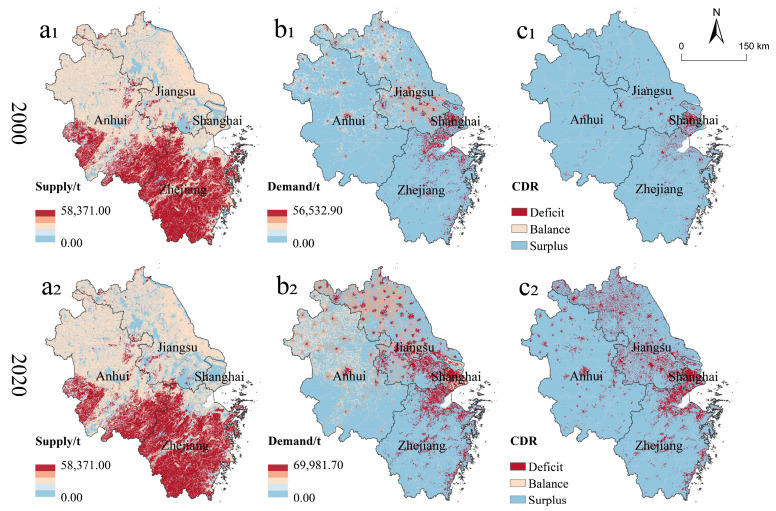
Spatial distribution of carbon storage supply (**a_1_**,**a_2_**), carbon storage demand (**b_1_**,**b_2_**) and CSDR (**c_1_**,**c_2_**) in YRD in 2000 (**upper** panel) and 2020 (**lower** panel), respectively.

In terms of spatial distribution (Figure 5), the CSDR of Shanghai varied from surplus to deficit with the CSDR falling from 0.14 to −0.27 during 2000–2020. The proportion of CSDR deficit areas in Shanghai was also the highest (47.82% in 2020), and nearly half of the areas showed imbalanced CSD. Although the CSDR of Jiangsu remained in surplus, it also declined from 0.86 in 2000 to 0.53 in 2020, indicating a serious risk of deficit. In 2020, 13.12% of CSDR deficit areas in Jiangsu were concentrated along Taihu Lake and the Yangtze River. Zhejiang has the largest forest ecosystem in YRD, which provides the strongest carbon storage function. However, the rapid increase of carbon storage demand (275.89%) since 2000 caused a decrease of its CSDR from 0.94 to 0.80. Geographically, 9.53% of the CSDR deficit areas in Zhejiang were located along the Hangzhou Bay, Fuchun River and southeast coastal areas. Anhui had the second largest carbon storage supply capacity with the highest CSDR in YRD; the relatively low rate and scale of urbanization made its carbon storage demand relatively low. But the expansion of urbanization still caused the CSDR of Anhui falling rapidly from 0.96 in 2000 to 0.87 in 2020. Here, deficit regions were mainly located along the Yangtze River, central and northern of Anhui.

### 4.2. Impact of Urbanization on CSD

#### 4.2.1. The Relationship between Urbanization and CSD

**Figure 6:**
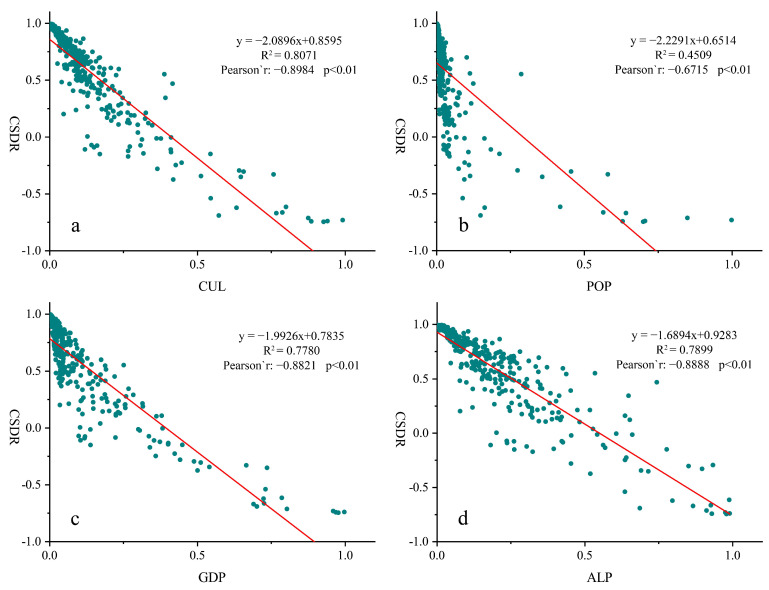
Linear regression model showing the CSDR dependency of integrated urbanization indicator CUL and each urbanization indicators in 2020.

The Pearson correlation analysis indicated that demographic urbanization, economic urbanization, spatial urbanization and CUL all had significant negative correlation with CSDR (*p <* 0.001) (Figure 6). The linear regression analysis revealed that CUL had a high explanatory rate for the change of CSDR ($R^{2}=0.8071$ and the slope is −2.0896), indicating that CSDR tended to be in deficit with the increase of CUL. Similar relationships could be found in terms of ALP ($R^{2}=0.7899$) and GDP ($R^{2}=0.7780$), representing the spatial and economic urbanization, respectively. It is obvious that economic urbanization had a much stronger influence than spatial urbanization on CSDR (their slope was −2.0896 and −1.6894 respectively). That means carbon emissions resulted from economic development contributed more to carbon storage demand. The explanatory rate of population urbanization to CSDR was relatively low, and the effect of population urbanization on CSDR showed a non-linear decline. Overall, economic urbanization made a major contribution to CSDR change in YRD.

#### 4.2.2. Strength of Urbanization Impact on CSDR

**Figure 7:**
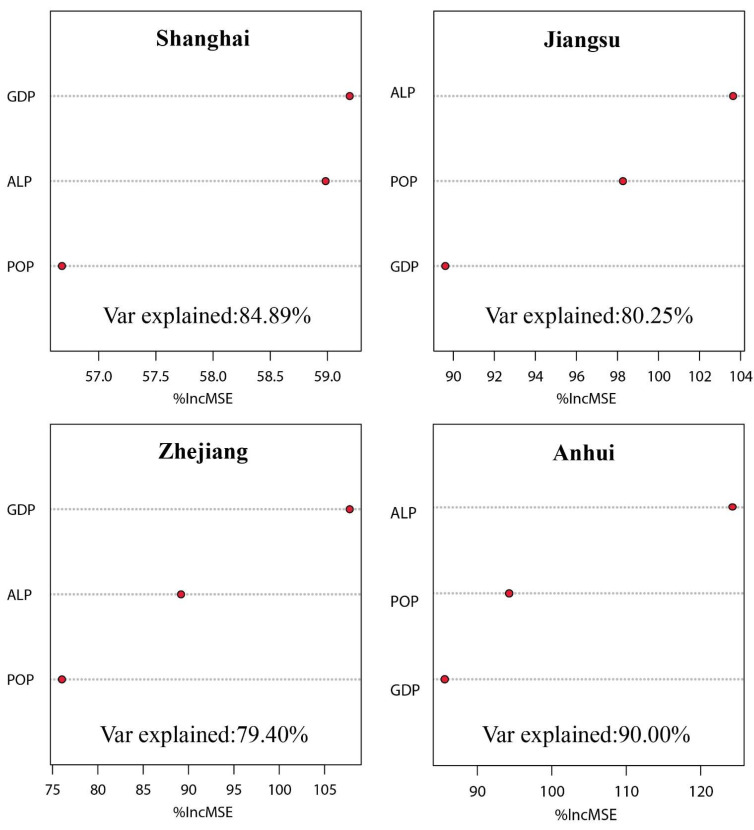
Sensitivity of CSDR to urbanization indicators in different regions in 2020.

CSDR was significantly influenced by economic urbanization and spatial urbanization (Figure 7), because all urbanization indicators had a high overall explanatory rate to CSDR with Var explained values higher than 70%. Specifically, CSDR of Shanghai and Zhejiang had a stronger sensitivity to economic urbanization. Shanghai is the economic center of YRD; the high intensity of economic activities had resulted in massive consumption of energy, and thus the region had higher carbon storage demands. The CSDR of Jiangsu and Anhui were much sensitive to spatial urbanization, where fast expansion of urban built-up areas changed the structure of natural ecosystems and impaired their carbon storage capacity.

### 4.3. CSD Response Measures

#### 4.3.1. Priority Conservation Areas for Carbon Storage Supply

Marxan model simulation results depicted that 8.60% of the area of YRD’s priority protected areas for carbon storage supply in the 2020 scenario were located in the western and southern of Anhui, and the western and southern of Zhejiang. Forest ecosystems in these areas were well preserved and had strong capacities for carbon storage. Besides, these areas were mainly hilly and mountainous terrain unsuitable for diverse human activities, so their level of urbanization and carbon storage demand were relatively lower. In the 2030 scenario, carbon storage supply priority reserves in YRD should expand to 12.58% of its total area to meet the carbon peak demand. Anhui and Zhejiang provinces are still responsible for providing most of the carbon storage. Jiangsu province and Shanghai have limited space suitable for carbon storage supply due to their low forest cover and high ecological conservation costs.

**Figure 8:**
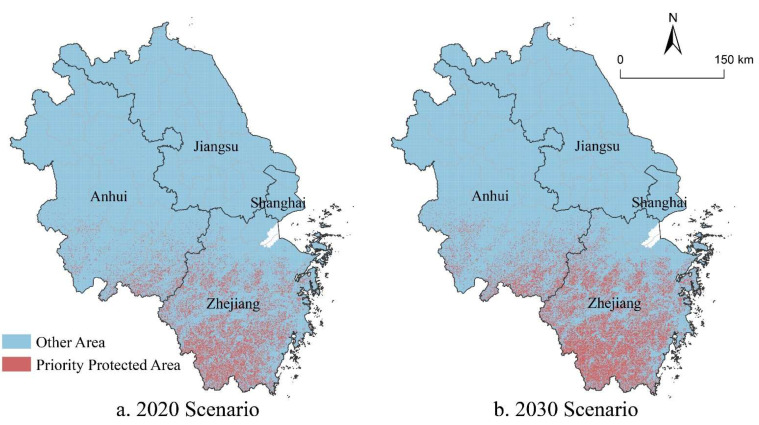
Distribution of priority conservation areas for carbon storage under different scenarios.

The priority areas in YRD were fragmentized under the influence of urbanization. In order to improve management efficiency, the priority conservation areas in YRD were designated in conjunction with the carbon storage supply priority areas and forest distribution (Figure 8). The carbon storage function priority reserve covered 33.98% of the total area of YRD, including the Dabie Mountains in western Anhui, the southern mountains of Anhui province, the eastern hilly mountains of Anhui, and the western and southern mountains of Zhejiang province. These priority conservation areas could provide 53.16% of the total carbon storage supply in YRD, however their average CUL combined was only 25.09%. Hence, it is possible to maintain the carbon security pattern of YRD with a relatively small ecological cost.

**Figure 9:**
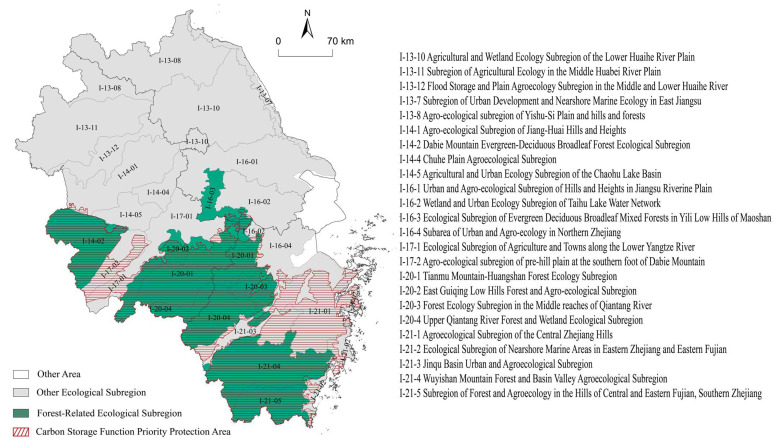
Comparison between the priority protected area for carbon storage function and the established ecological function zoning scheme in YRD.

Comparing the priority carbon storage function reserves with the established ecological function zoning scheme (data from the China Ecological Function Zoning Database: https://www.ecosystem.csdb.cn/ecoass/ecoplanning.jsp, accessed on 27 June 2022), the area of subregions related to forest protection is 100,600 km^2^, accounting for 28.09% of the total area of YRD. The area of priority carbon storage reserves is 24,300 km^2^, which is larger than the forest-related subregions in existing ecological function zoning scheme. The additional priority carbon storage reserves are mainly distributed along the Yangtze River in southwest of Anhui province and the hilly area in central of Zhejiang province. In the existing ecological function zoning scheme, these areas were designated as agroecological subregions (Figure 9, I-17-1, I-17-2, I-21-1). Additionally, the Yili low-hill mixed-evergreen deciduous broad-leaved forest ecological subregion (Figure 9, I-16-3) in Maoshan was excluded because of its intensive human activities and high level of urbanization.

#### 4.3.2. Construct Carbon Compensation Scheme

Due to the regional differences of CSD and development positioning among different regions in YRD, it is necessary to build a carbon compensation scheme in the context of regional integration, and to coordinate the contradictory relationship between socio-economic development and ecological protection among these regions. For example, the CSDR of Shanghai was in deficit, but it is one of the economic centers in China and still needs to focus on socio-economic development in the future; the south of Anhui had a stronger ecological foundation but less urbanization, which can be advantageous for ecological conservation.

**Figure 10:**
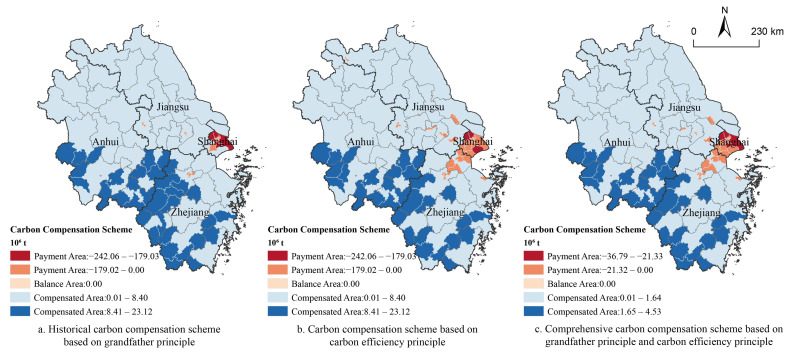
Carbon compensation scheme under different principles.

According to the grandfather principle, a total of 19 counties in the YRD were historical carbon payment areas during 2000–2020 (Figure 10a), with a total of 111,860.35 million tons of historical carbon credits to be paid. Geographically, 12 of 19 historical carbon payment counties were concentrated in Shanghai with a total of 109,456.02 million tons of historical carbon credits to be compensated. In terms of historical carbon compensation, Zhejiang province and Anhui province were the most important historical carbon compensation areas, accounting for 41.78% and 40.29% of YRD’s total historical carbon compensation, respectively.

Based on the carbon efficiency principle, there were 37 counties in the YRD defined as carbon payment areas in 2020 (Figure 10b) with a total of 136,427,600 tons of carbon emission credits. These counties mainly concentrated in Shanghai and Hangzhou Bay coastal area. Specifically, Shanghai accounted for 79.63% of the total carbon payment in YRD. Zhejiang province and Anhui province were still the main carbon compensation areas, accounting for 41.48% and 46.27% of the total carbon compensation in YRD, respectively.

Integrating the grandfather principles and carbon efficiency principles, 24 counties in YRD were identified as carbon payment zones in 2020 (Figure 10c), with a total of 210,399,800 tons of carbon credits to be paid. The number of carbon payment zones had reduced by 13. These zones were carbon payment zones in 2020, but historical carbon compensation zones before 2020, who could enjoy historical carbon compensations; their assessed annual average historical carbon compensations were higher than the carbon payments required in 2020. These carbon payment zones were mainly located in Shanghai and along the coasts of Hangzhou Bay, including 15 counties in Shanghai, because Shanghai had a high intensity of spatial urbanization without sufficient ecological space for carbon storage. Shanghai had an early economic development and a high level of economic urbanization, and long-term economic activities had caused a large amount of CO_2_ emissions. Therefore, Shanghai was responsible for the largest share of historical carbon emissions. Some counties in Hangzhou and Jiaxing of Zhejiang province and Nanjing and Wuxi of Jiangsu province were classified as historical carbon payment areas. In particular, the overall carbon storage supply in Hangzhou was much higher than carbon storage demand, but the concentrated distribution of highly urbanized areas in Hangzhou made carbon storage demand highly concentrated in city center. Zhejiang province and Anhui province remained to be the most important carbon compensation zones accounting for 41.66% and 42.25% of the total carbon compensations in YRD, respectively.

#### 4.3.3. Identify Thresholds of YRD Urbanization

The fitted regression prediction results showed (Table 1) that YRD would suffer a high risk of imbalance of CSD when the population density (POP) exceeds 5674.70 people/km^2^, the GDP density (GDP) exceeds 0.3807 billion yuan/km^2^ and the urban built-up area ratio (ALP) exceeds 0.5289. Overall, the CSD balance of YRD will reach its threshold when the CUL is over 0.3082.

**Figure 11:**
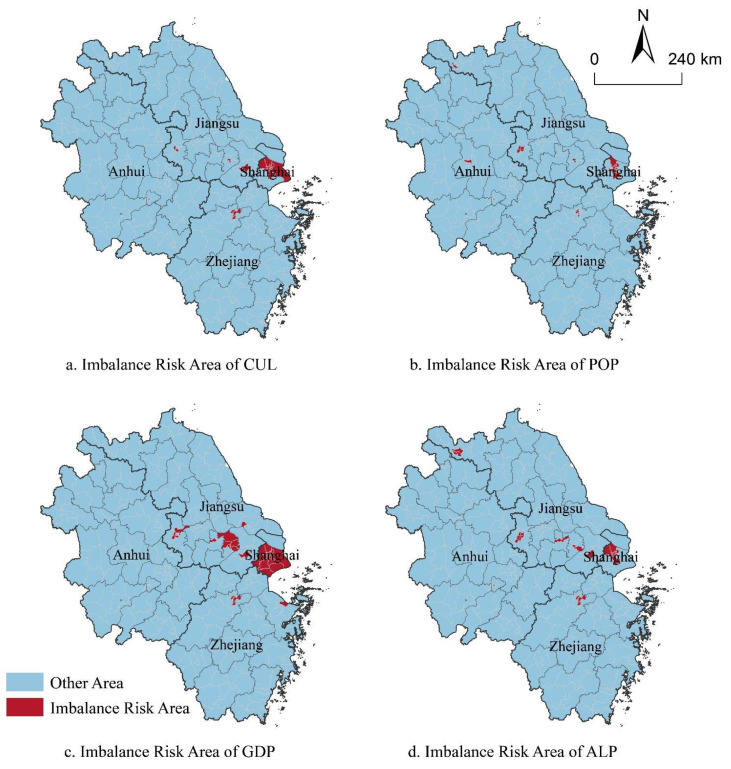
Risk areas of imbalanced CSD under different urbanization drivers.

In general (Figure 11), a total of 21 counties in YRD had exceeded the CUL threshold and were confronted with the risk of imbalance of CSD. The POP of 20 counties, the GDP of 33 counties, and the ALP of 29 counties in YRD had exceeded their thresholds in 2020. The risk areas were relatively concentrated, among which Shanghai had the largest number of risk areas, indicating that Shanghai might undertake enormous pressure to independently achieve the carbon neutrality target before 2060. Besides, the centers of some large cities in Jiangsu, Zhejiang and Anhui province were also faced with high risk of imbalanced CSD.

## 5. Discussion

### 5.1. Comparison with Previous Studies

Mega-urban agglomeration is facing increasing pressure on resources and ecology in the development process [2]. We have therefore constructed a SES framework that combines the ES cascade with the DPSIR model. The framework allows for a comprehensive analysis of the impact of urbanization on sustainable development, and it considers responsive management measures for urbanization drivers, ecological subsystems and social subsystems, facilitating the reconciliation of urbanization with the ecological environment. Using YRD as an example, we applied the SES framework to demonstrate the impact of urbanization on CSD and proposed countermeasures to facilitate YRD to achieve their carbon peaking and carbon neutrality targets. Consistent with former research [15,48,49,50], we found that carbon storage supply decreased and carbon storage demand increased in YRD with rapid urbanization, so it is necessary to strengthen the construction of a priority conservation area for carbon storage supply, while constructing a scientific carbon compensation scheme and setting reasonable urbanization development goals. This study revealed the coupling mechanism between the ecosystem process following the laws of nature and the urbanization development process following the laws of humanity, explored the interaction between urbanization and CSD in mega-urban agglomeration, proposed targeted ecological management measures to make up for the lack of research on the response of CSD to urbanization, promoted the sustainable development of urbanization and CSD in YRD, and provided a reference for other mega-urban agglomeration to carry out research on the interaction between urbanization and CSD.

### 5.2. Implication for Ecological Management

Although the total carbon storage supply still meets the demand, spatial mismatched CSD is quite common in YRD, especially in urbanized areas. Therefore, it is essential to provide a clear map of carbon storage surplus and deficit in YRD to managers, and following aspects might be considered for ecological management.

**(1) Build a carbon ecological security pattern and regulate spatial mismatched CSD**. In the context of integrated development in YRD, the constraints of administrative boundaries could be broken down to achieve synergistic management. Identify ecological sources and sinks based on carbon storage surplus and deficit and construct ecological safe patterns through ES flow analysis. On the one hand, it is urgent to protect important ecological spaces through establishing nature reserves; on the other hand, we need to improve the connectivity of ecological spaces through ecological restoration, promote CSD flow, and realize the optimal spatial deployment of CSD.

**(2) Improve CSD through opening the source and regulating the flow**. Ecological management measures should be developed in terms of both ‘open-source’ and ‘cost-cutting’. Open-source focuses on increasing carbon storage supply by restoring ecologically occupied or damaged land. Cost-cutting emphasizes on improving the efficiency of carbon emissions, which means reducing carbon storage demand by reducing carbon emissions while ensuring economic development. Hence, we need to promote energy reform by seeking new renewable and clean energy sources to replace traditional fossil fuels and strengthen research and development of energy storage technologies to solve the current instability in water and wind power generation. Aside from those concerns, we should actively promote industrial structure optimization and transformation and upgrade technologies to improve the efficiency of energy use per unit.

**(3) Coordinate ecological protection with socio-economic development and clarify the ecological management priorities**. Shanghai is a highly urbanized city, but one lacking in sufficient ecological land to enhance carbon storage supply. Therefore, Shanghai should optimize its carbon storage supply through strengthening green infrastructure development, while improving carbon efficiency through industrial restructuring and upgrading. Externally, it is possible to compensate its carbon emissions by optimizing the ecological security pattern, for example, building a forest belt around Shanghai. Jiangsu province has relatively poor ecological resources but a high level of urbanization. It is necessary to limit the speed and expansion of urbanization and promote its quality. Furthermore, through increasing construction of green infrastructure and ecological restoration it is possible to improve its capacity of carbon storage supply. Finally, a green-low-carbon economy should be vigorously developed to reduce energy consumption and improve ecological efficiency. Zhejiang province has excellent ecological conditions. Except for strict ecological protection, they should strive to explore the ecological value conversion model and transform the advantages of ecological resources into ecological and economic values. The above-mentioned measures are also applicable to Anhui province, considering the spatial divergence between urbanization development and CSD. 

**(4) Promote quality of urbanization and rationalize CSD balance**. YRD will continue to experience rapid urbanization for a long time. It is impossible to simply control the speed and scale of urbanization to balance CSD, but leaders should strive to promote high-quality urbanization. In terms of carbon storage supply, a certain amount of compensation could be taken from the benefits of urbanization to strengthen ecological construction and enhance carbon storage supply capacity. As for the carbon storage demand, the ecological efficiency should be improved through scientific and technological innovation and industrial upgrading, and the energy consumption structure should be reformed. 

### 5.3. Limitations

There are some limitations in this research. Firstly, CSD in YRD was influenced by urbanization, and we promoted response measures based on static analysis. However, the dynamics of CSD in the SES system were a complex process; it is necessary to apply related ES flow theory to explore CSD regulation. Secondly, we selected 2000–2020 as the historical responsibility period for theoretical research. However, the definition of the historical responsibility period for carbon emissions should be decided by governments through negotiation and game theory. Finally, this study only measured the total amount of carbon offsets without discussing carbon compensation. Current research mainly calculated carbon compensation based on their market values [51,52]. But this approach was difficult to apply in practice, as the amount of carbon compensation per unit need to be negotiated by governments. Hence, further research could make breakthroughs in these aspects. 

## 6. Conclusions

In this study, we measured CUL and spatial-temporal characteristics of CSD based on multi-source datasets, and explored the impact of urbanization on CSD, and then promoted relative measures in response to urbanization, ecological and social subsystems from a supply and demand perspective in the context of carbon target. The following conclusions were obtained:

(1) The average CUL increased by 100.00% during 2000–2020 due to rapid urbanization in YRD. Carbon storage supply in YRD decreased by 2.75%, while carbon storage demand increased by 226.45%. The total of carbon storage supply in YRD was greater than carbon storage demand, but the CSDR still decreased by 19.39%. In addition, there was a significant spatial mismatch between urbanization and CSDR in YRD.

(2) POP, GDP, ALP and CUL of YRD had significant negative correlations with CSDR. CSDR was obviously sensitive to GDP in Shanghai and Zhejiang; but is sensitive to ALP in Jiangsu and Anhui.

(3) To compensate carbon emissions, 8.60% and 12.58% of the total area of YRD should be established as carbon storage supply priority reserves in the 2020 and 2030 scenarios, respectively, to sustain its carbon storage supply capacity. In addition, to strengthen the protection of the carbon storage function, and maintain the integrity and continuity of the ecosystem, the carbon storage protection zones were designated on the base of carbon storage supply priorities, which accounted for 33.98% of the total area of YRD.

(4) Integrating the grandfather principle and the carbon efficiency principle, a total of 24 counties in YRD would be carbon payment zones in 2020, with 210,399,800 tons of carbon emissions to be paid. Shanghai and its surrounding areas were the main carbon payment areas; Anhui and Zhejiang province were the most important carbon compensation areas. 

(5) When POP exceeded 5674.70 people/km^2^, GDP exceeded 0.3807 billion yuan/km^2^, ALP exceeded 0.5289 and CUL exceeded 0.3082, YRD might suffer high risk of imbalance of CSD.

## Figures and Tables

**Table 1:** Thresholds of urbanization development using regression prediction model.

Index	Regression Prediction Model	R^2^	Threshold
POP	y=−0.281ln(x)+2.4289	0.7337	5674.70 (people/ km^2^)
GDP	$y=1.2297x^{2}-2.7425x+0.8659$	0.8330	0.3807 (Billion/km^2^)
ALP	$y=0.5112x^{2}-2.1266x+0.9817$	0.7959	0.5289
CUL	y=−2.8183x+0.8686	0.8070	0.3082

## Data Availability

The data presented in this study are available on request from the corresponding author.

## Supplementary Materials

The following supporting information can be downloaded at: https://www.mdpi.com/article/10.3390/ijerph192113768/s1, Methods S1: GDP density mapping; Methods S2: Carbon density correction; Methods S3: Carbon storage demand measurement and spatial mapping; Methods S4: Random Forest: Importance Analysis of Influencing Factors ; Methods S5: Identification of priority conservation areas for carbon storage supply using Marxan model; Methods S6: Carbon payment correction coefficient and carbon compensation correction coefficient; Table S1: Carbon intensity values for each land use type in China in the previous research ; Table S2: YRD carbon density correction value of various land use types. References [39,40,41,42,43,44,53,54,55,56,57,58,59,60,61] are cited in the supplementary materials.

## Abbreviations

CSD	carbon storage supply and demand
ES	ecosystem services
YRD	Yangtze River Delta Region
CSDR	carbon storage supply and demand ratio
SES	social-ecological systems
CUL	combined urbanization level
POP	population density
GDP	GDP density
ALP	artificial land proportion
