# Supplementary material for: The Impact of Urbanization on the Relationship between Carbon Storage Supply and Demand in Mega-Urban Agglomerations and Response Measures: A Case of Yangtze River Delta Region, China
Source: Int J Environ Res Public Health. 2022 Oct 23;19(21):13768. doi: 10.3390/ijerph192113768 (PMC9658919; doi:10.3390/ijerph192113768)
Supplement: Supplementary file 1 [file ijerph-19-13768-s001.zip › ijerph-1909285-supplementary.pdf]

International Journal of
Environmental Research and Public Health

MDPI

The Impact of Urbanization on the Relationship between Carbon Storage Supply and Demand in Mega-Urban Agglomerations and Response Measures: A Case of Yangtze River Delta Region, China

Yinan Yang [1,†], Jing Li [1,2,†], Li Wang [1,3], Zihao Wang [1], Yun Ling [1], Jialong Xu [1], Chenxin Yao [1], Yiyan Sun [1], Yuan Wang [1,3,*] and Lixia Zhao [4,5,6,*]

[1] School of Geography and Tourism, Anhui Normal University, Wuhu 241002, China
[2] China Railway Shi Dai Architectural Design Institute Co. Ltd., Wuhu 241000, China
[3] Neweco Design Co. Ltd., Shanghai 200433, China
[4] East China Sea Ecological Center, Ministry of Natural Resources (MNR), Shanghai 201206, China
[5] Key Laboratory of Marine Ecological Monitoring and Restoration Technology, Ministry of Natural Resources (MNR), Shanghai 201206, China
[6] Key Laboratory of Ocean Space Resource Management Technology, Ministry of Natural Resources (MNR), Hangzhou 310012, China
* Correspondence: oneyuan1216@gmail.com (Y.W.); zhaolixia@ecs.mnr.gov.cn (L.Z.)
† These authors contributed equally to the work.

Supplementary Materials:

Methods Captions

Methods S1 GDP density mapping

Methods S2 Carbon density correction

Methods S3 Carbon storage demand measurement and spatial mapping

Methods S4 Random Forest: Importance Analysis of Influencing Factors

Methods S5 Identification of priority conservation areas for carbon storage supply using Marxan model

Methods S6 Carbon payment correction coefficient and carbon compensation correction coefficient

Table Captions

Table S1 Carbon intensity values for each land use type in China in the previous research

Table S2 YRD carbon density correction value of various land use types

Methods S1 GDP density mapping

The GDP density data is based on the inverse of the regression relationship between GDP statistics and land use and night lighting data for each city in the Yangtze River Delta region. The output value of the primary industry is equally distributed among the land use types of arable land, forest land, grassland and water. The output value of secondary and tertiary industries is then inverted by constructing a fitted regression model with nighttime lighting data on land use types in urban areas [1].

$$G_{ie} = -0.0196x^2 + 1412.4x + 3000000(R^2 = 0.8067) \quad (S1)$$

where G_{ie} is the estimated output value of secondary and tertiary production from the fit for site i; and x is the night-time light value.

Linear correction of the fitted secondary and tertiary output values using actual secondary and tertiary output statistics.

$$G_{iC} = G_{ie} \times (\frac{G_{is}}{G_{ie,all}}) \quad (S2)$$

where G_{ic} is the linearly corrected raster second and third output value of place i; G_{is} is the total statistical value of second and third output value of place i; $G_{ie,all}$ is the estimated total value of second and third output value fitted to the Yangtze River Delta region.

Methods S2 Carbon density correction

Carbon density data were obtained from the previous research on carbon density by land use type in China[2-5] (Table 1S), and there are some differences in carbon density between different regions due to different geographical factors. Previous studies have shown that both biomass carbon density and soil carbon density show significant correlation with mean annual precipitation and weak correlation with mean annual temperature [6-8]. Therefore, the biomass carbon density and soil carbon density in the Yangtze River Delta region were corrected based on the average annual precipitation [6,9].

$$C_{AD} = 25.8 + 0.071 \times MAP(R^2 = 0.67) \quad (S3)$$

$$C_{BD} = 8.3 + 0.013 \times MAP(R^2 = 0.53) \quad (S4)$$

$$C_{SP} = 3.3968 \times MAP + 3996.1(R^2 = 0.11) \quad (S5)$$

Where C_{AD} is the above-ground biomass carbon density (t/hm^2) corrected for average annual precipitation, C_{BD} is the below-ground biomass carbon density (t/hm^2) corrected for average annual precipitation, C_{SP} is the soil carbon density (t/hm^2) corrected for average annual precipitation, and MAP is the average annual precipitation.

$$K_{AD} = \frac{C_{AD}^{`}}{C_{AD}^{``}} \quad (S6)$$

$$K_{BD} = \frac{C_{BD}^{`}}{C_{BD}^{``}} \quad (S7)$$

$$K_{SP} = \frac{C_{SP}^{`}}{C_{SP}^{``}} \quad (S8)$$

Where K_{AD}, K_{BD} and K_{SP} are above-ground biomass carbon density correction factors, below-ground biomass carbon density correction factors and soil carbon density correction factors, respectively. $C_{AD}^{`}$ and $C_{AD}^{``}$ are the above-ground biomass carbon density data obtained at the Yangtze River Delta regional scale and the national scale based on the correction factor for average annual precipitation, respectively; $C_{BD}^{`}$ and $C_{BD}^{``}$ are the below-ground biomass carbon density data obtained at the Yangtze River

Delta regional scale and the national scale based on the correction factor for average annual precipitation, respectively; $C_{SP}^{`}$ and $C_{SP}^{``}$ are the soil carbon density data at the Yangtze River Delta regional scale and national scale respectively, corrected by the average annual precipitation factor. The corrected carbon density values for the Yangtze River Delta region are shown in Table 2S.

Methods S3 Carbon storage demand measurement and spatial mapping

The accounting of the demand for carbon storage services is accounted for using the end-use energy consumption-based carbon emissions methodology proposed by the IPCC [10]. The calculation formula is as follows.

$$CP = CE = \sum E \times NCV \times EF \quad \text{(S9)}$$

Where *CP* is the total demand for carbon storage services (tons); *CE* is the total carbon emissions (tons); *E* is the total energy consumption (standard coal); *NCV* is the net calorific value of energy (*TJ/t*), from the China Energy Statistics Yearbook; *EF* is the emission factor of CO_2 (*t/TJ*), from the carbon emission factor coefficients provided by the IPCC.

Carbon emissions are mapped based on the relationship between land use types and carbon emissions from the primary, secondary and tertiary sectors, with carbon emissions from the primary sector allocated equally to arable land. The carbon emissions from secondary and tertiary industries were inferred by constructing a regression model between nighttime lighting and carbon emissions on urban, rural, industrial, mining and residential land use types. The total carbon emissions of each industry were determined according to the proportion of total energy consumption of primary, secondary and tertiary industries in the Yangtze River Delta region.

The inverse model of nighttime lighting and carbon emissions for the secondary and tertiary industries is as follows [11].

$$lnC_f = 0.36lnx + 2.73 \ (R^2 = 0.87) \quad \text{(S10)}$$

where C_f is the estimated raster carbon emissions (*t*); x is the nighttime light value. and C_f is corrected based on the actual total carbon emissions (*CD*) from the secondary and tertiary sectors accounted for by energy consumption.

$$C_m = C_f \times (CD/C_{f,all}) \quad \text{(S11)}$$

where C_m is the corrected raster carbon emissions value (t); $C_{f,\text{all}}$ is the total estimated value of all raster carbon emissions inversions.

Methods S4 Random Forest: Importance Analysis of Influencing Factors

Random Forest (RF) is a decision tree based machine learning algorithm that can be used for classification and regression analysis [12]. The Random Forest regression model can explain the degree of influence of the independent variable on the dependent variable, where the sensitivity represents the contribution of the independent variable to the accuracy of the Random Forest regression model, i.e., the importance of the independent variable to the model. Therefore, using the carbon storage supply-demand ratio (CSDR) as the dependent variable and POP, GDP, and ALP as the independent variables, the sensitivity characteristics of CSDR to urbanization were analyzed by the random forest regression model to reflect the importance of each urbanization indicator that affects the change of CSDR. The sensitivity analysis based on the Random Forest regression model was conducted in R Studio software using the Random Forest package, using IncMSE values to characterize the sensitivity characteristics, IncMSE refers to the proportion of increase in MSE (mean square error), and the higher the IncMSE value, the more sensitive the dependent variable is to the independent variable, i.e., it represents the more important independent variable. The relevant calculation formula is as follows.

$$\text{MSE} = \frac{\sum_{i=1}^{n}(\hat{y}_i - y_i)^2}{n} \quad \text{(S12)}$$

$$\text{IncMSE} = \frac{MSE_{after-}MSE_{before}}{MSE_{before}} \quad \text{(S13)}$$

where *n* is the sample size, y_i is the actual value of sample *i*, and $\hat{y}_i$ is the predicted value of sample i. MSE_{before} is the initial MSE of the model, and MSE_{after} is the MSE of the model after replacing the independent variables with a set of random values.

In random forest regression models, Var explained (percentage variance explained) is used to measure the degree of match between the predicted and actual values of the model, and a larger Var explained value indicates a more accurate prediction of the model. The relevant calculation formula is as follows.

$$\text{Var explained} = 1 - \frac{\sum_{i=1}^{n}(\hat{y}_i - y_i)^2}{\sum_{i=1}^{n}(y_i - \bar{y})^2} \quad \text{(S14)}$$

where *n* is the sample capacity; y_i is the actual value of sample i; $\hat{y}_i$ is the predicted value of sample i; and $\bar{y}$ is the average of the actual value of sample *i*.

Methods S5 Identification of priority conservation areas for carbon storage supply using Marxan model

1. Modeling Principle

The Marxan model is a conservation planning decision aid model developed based on the theory of systematic conservation planning [13]. It calculates the irreplaceability values of planning units through simulated annealing algorithm and identifies planning units with high irreplaceability as priority conservation areas [14]. Based on this model, priority conservation areas for carbon storage supply that meet the ecological conservation objectives with relatively low ecological conservation costs can be identified.

The main formula of the Marxan model is as follows [15].

$$\text{minimize} \sum_{i}^{N_s} x_i c_i + b \sum_{i}^{N_s} \sum_{h}^{N_s} x_i (1 - x_h) cv_{ih} \quad \text{(S15)}$$

However, all conservation objectives must be met.

$$\sum_{i}^{N_f} x_i r_{ij} \geq T_j \forall j \quad \text{(S16)}$$

where x_i is either 0 or 1.

$$x_i \in \{0,1\} \forall i \quad \text{(S17)}$$

where x_i is 1 if the planning unit is selected as a priority conservation area, and 0 if the planning unit is not selected as a priority conservation area. In Equation (A.15), N_s is the number of planning units; c_i is the cost of planning unit i;

cv_{ih}indicates the connection cost between planning unit i and planning unit h; b value is the boundary multiplier (or boundary length modifier, BLM), which is a custom parameter, and the higher the b value, the more compact the planning unit. In Equation (A.16), T_j is the protection target of a specific protection object. r_{ij} is the frequency of occurrence of protection feature j in planning cell i; N_f is the number of protection features.

To be able to compare different solutions and thus determine a better solution, Marxan integrates the objective function and the constraint function into one objective function, transforming the constraint function into an additional penalty think to solve the problem. The mathematical expression takes the following form.

$$\sum_i^{N_s} x_i c_i + b\sum_i^{N_s}\sum_h^{N_s} x_i(1-x_h)cv_{ih} + \sum_j^{N_f} FPF_j\, FR_j H(s)(\frac{s}{T_j}) \quad \text{(S18)}$$

Where FPF_j and FR_j distributions are feature penalty factors and protection object characteristics, and FPF_j is a scaling factor that determines the relative importance of the protection object that satisfies feature j. FR_j is calculated as the protection cost of the protection object that satisfies feature j. The difference s is the amount of the target of protection that was not reached, with the following formula.

$$s = T_j - \sum_i^{N_f} xr_{ij} \quad \text{(S19)}$$

The Heaviside function H(s) is a step function that takes the value 0 when s⩾0 and 1 otherwise. The feature-specific parameter T_j is the target representation of feature j; $(\frac{s}{T_j})$ is a measure of the underrepresentation of feature j, which is reported as a ratio equal to '1' when feature j is not represented in the configuration, and close to '0' when the level of representation is close to the target amount. The Heaviside function ensures that the entire equation is zero when the representation is greater than the target amount.

2. Operation process

(1) Determine the planning units and boundaries: Using the "Create Fishing Network" tool in ArcGIS, the Yangtze River Delta region was divided into 360061 1km1km grids as planning units, and the Yangtze River Delta region was used as the planning area and the boundaries were defined.

(2) Identify the conservation targets: The purpose of this part of the study is to identify the priority conservation areas for carbon storage supply that are sufficient to offset the demand for carbon storage, so the conservation targets are carbon storage supply.

(3) Calculate the cost of the planning unit: In order to ensure that the identified priority conservation areas for carbon storage supply can maintain a stable supply capacity, it is necessary to implement more stringent protection measures for the priority conservation areas. In order to avoid the remaining irrelevant activities within the priority conservation areas, it is necessary to seek areas with low human activities as priority conservation areas as much as possible, so the comprehensive urbanization level (CUL) is used as the conservation cost.

(4) Determination of conservation objectives： In terms of the conservation target, two scenarios were set based on the current study year (2020) and the year in which the carbon peak target is scheduled to be achieved (2030), and the proportion of carbon storage supply to the total carbon storage supply in YRD when the carbon storage supply and demand reach the equilibrium point in each scenario was set as the conservation target. Firstly, according to the results of the previous analysis, the proportion of carbon storage supply that

needs to be conserved when carbon storage supply and demand reach the equilibrium point in 2020 is 0.13. Secondly, based on the changes in carbon storage supply and demand in YRD during the period 2000-2020, a stepwise regression method was used to find the best-fitting regression forecast model to predict carbon storage supply and demand in YRD in 2030, so as to obtain the ratio of carbon storage supply to the total carbon storage supply in YRD that needs to be protected when carbon storage supply and demand reach the equilibrium point in 2030 as 0.22. The conservation targets for the 2020 scenario and the 2030 scenario are therefore 0.12 and 0.22 respectively. The regression forecasting models for carbon storage supply (y_supply) and demand (y_demand) are as follows.

$$y_{supply} = -0.7099x + 116.44 \quad (R^2 = 0.9945) \tag{S20}$$

$$y_{demand} = 3.3486x + 2.1611 \quad (R^2 = 0.9847) \tag{S21}$$

(5) Priority conservation area identification: we obtained irreplaceability value of each planning unit after running the Marxan model 10,000 times. Irreplaceability value refers to the number of times a planning unit is selected as a priority conservation area, and higher value indicates more im-portant the conservation area is. Combining irreplaceability values with conservation objectives, the priority conservation areas, consisting of planning units with irreplace-ability values greater than 2,788 and 3,421 times in the 2020 and 2030 scenarios re-spectively, were set to be thresholds to achieve balanced CSD in YRD.

Methods S6 carbon payment correction coefficient and carbon compensation correction coefficient

The carbon payment correction factor CR based on carbon efficiency is shown in equation (A.22) and the carbon compensation correction factor PR based on carbon efficiency is shown in equation (A.23).

$$CR_i = \frac{a_{n,cr} b_{n,cr} c_{n,cr} X_{n,cr}}{\sum a_{n,cr} b_{n,cr} c_{n,cr} X_{n,cr}} \tag{S22}$$

Where CR_i is the carbon payment correction coefficient of area i; $a_{n,cr}$, $b_{n,cr}$ and $c_{n,cr}$ are the standardised values (positive indicators) of carbon emissions per unit area, carbon emissions per unit GDP and carbon emissions per unit population respectively, standardised using the Max-Min method, see equation (A.24); $X_{n,cr}$ is the actual carbon payment of area i.

$$PR_i = \frac{a_{n,pr} b_{n,pr} c_{n,pr} X_{n,pr}}{\sum a_{n,pr} b_{n,pr} c_{n,pr} X_{n,pr}} \tag{S23}$$

Where PR_i is the carbon compensation correction factor for area i; $a_{n,pr}$, $b_{n,pr}$ and $c_{n,pr}$ are the standardized values of carbon emissions per unit area, carbon emissions per unit GDP and carbon emissions per unit population respectively (negative indicators), which are standardized using the Max-Min method, see equation (A.25); $X_{n,pr}$ is the actual carbon compensation for area i.

$$\theta_{std} = \frac{\theta_{ij} - Min_{ij}}{Max_{ij} - Min_{ij}} \quad \text{Positive indicators} \tag{S24}$$

$$\theta_{std} = \frac{Max_{ij} - \theta_{ij}}{Max_{ij} - Min_{ij}} \quad \text{Negative indicators} \tag{S25}$$

where θ_{std} is the normalized value, θ_{ij} is the original series, and Max_{ij} and Min_{ij} are the maximum and minimum values in the original series.

Table S1 Carbon intensity values for each land use type in China in the previous research

Land Use Type	Aboveground biomass (t/hm^2)	Belowground biomass (t/hm^2)	Soil (t/hm^2)	Death organic matter (t/hm^2)
Cropland	5.7	80.7	108.4	9.82
Woodland	142.4	115.9	129.2	14.11
Grassland	3.4	2.7114	99.9	7.28
Waters	0.3	0	0	0
Urban land	0	0	78	0
Unused land	1.3	0	0	0

Table S2 YRD carbon density correction value of various land use types

Land Use Type	Aboveground biomass (t/hm^2)	Belowground biomass (t/hm^2)	Soil (t/hm^2)	Death organic matter (t/hm^2)
Cropland	9.11	118.53	144.08	9.82
Woodland	227.64	170.23	171.73	14.11
Grassland	5.44	3.98	132.78	7.28
Waters	0.48	0.00	0.00	0.00
Urban land	0.00	0.00	103.68	0.00
Unused land	2.08	0.00	0.00	0.00

References

1. Hlab, C., Zga, B., Jwa, B., Zca, B., 2020.GDP spatialization in ningbo city based on NPP/VIIRS night-time light and auxiliary data using random forest regression. Adv. Space Res. 65(1), 481-493. DOI:10.1016/j.asr.2019.09.035.
2. Zhu, C., Zhao, S. Q., Zhou, D. C., 2012. Organic carbon storage in urban built-up areas of china in 1997-2006. Chin J Appl. Ecol. 23(5), 1195-1202. (In Chinses) DOI:10.13287/j.1001-9332.2012.0165.
3. Li, K. R., Wang, S. Q., Cao, M. K., 2003. Vegetation and soil carbon storage in China. Sci. Sinica(Terrae). 33(1),72 - 80. (In Chinses).
4. Piao, S. L., Fang, J. Y., He, J.S., et al., 2004. Spatial Distribution of Grassland Biomass in China. Acta Phyto. Sinica. 2004,28(4):491-498. (In Chinses).
5. Yang, J., 2021.Study on Grassland Ecosystem Service and Its Trade-off and Synergy in The Yellow River Basin. Gansu Agricultural University. (In Chinses) DOI:10.27025/d.cnki.ggsnu.2021.000048.
6. Chen, G. S., Yang, Y. S., Xie, J.S., et al., 2007. Total belowground carbon allocation in China`s forests. Acta Ecol. Sinica. 27(12):5148-5157.(In Chinese).
7. Alam, S.A., Starr, M., Clark, B.J.F., 2013. Tree biomass and soil organic carbon densities across the Sudanese woodland savannah: a regional carbon sequestration study. J. Arid Environ. 89, 67–76. DOI:10.1016/j.jaridenv.2012.10.002.
8. Giardina, C.P., Ryan, M.G., 2000. Evidence that decomposition rates of organic carbon in mineral soil do not vary with temperature. Nature. 404, 858–861. DOI:10.1038/35009076.
9. Guo, Y., Peng, C., Trancoso, R., et al., 2019. Stand carbon density drivers and changes under future climate scenarios across global forests. Forest Ecol. Manag. 49, 117463. DOI:10.1016/j.foreco.2019.117463.
10. IPCC,2006. IPCC guidelines for national greenhouse gas inventories. Institute for Global Environmental Strategies, Japan.
11. Pan, J. H., Zhang, Y. N., 2021. Spatiotemporal patterns of energy carbon footprint and decoupling effect in China. Acta Geogr. Sinica. 76(1), 206 - 222. (In Chinses) DOI: 10.11821/dlxb202101016.
12. Breiman, 2001. Random forests. Mach Learn. 45(1):5-32.
13. Ball, I.R., 2009. Possingham,Watts M.Marxan and relatives: Software for spatial conservation prioritization. Oxford Univ. Press, Oxford, UK.
14. McDonnell, M.D., Possingham, H., Ball, I.R., 2002. Cousins E. Mathematical models for spatially cohesive reserve design. Environ. Model. Assess. 7: 107– 114.
15. Norma, S.S., Alessia, K., Edward, T.G., et al., 2021. Possingham & Jennifer McGowan Marxan User Manual [EB/OL].2020.https://marxansolutions.org/software/.
